# What do multiple sclerosis patients and their caregivers perceive as unmet needs?

**DOI:** 10.1186/1471-2377-13-177

**Published:** 2013-11-15

**Authors:** Lorena Lorefice, Gioia Mura, Giulia Coni, Giuseppe Fenu, Claudia Sardu, Jessica Frau, Giancarlo Coghe, Marta Melis, Maria Giovanna Marrosu, Eleonora Cocco

**Affiliations:** 1 Multiple Sclerosis Center, University of Cagliari, via Is Guadazzonis, 2, Cagliari 09126, Italy; 2 Consultation Liaison Psychiatric Unit at the University Hospital of Cagliari, University of Cagliari and AOU, Cagliari, Italy; 3 Department of Public Health, University of Cagliari, Cagliari, Italy

## Abstract

**Background:**

Multiple sclerosis (MS) has a major impact on the physical, psychological and social life of patients and their families. The aim of this study was to evaluate the different perceptions of patients and caregivers about management of MS, particularly about the same items, to gather information to ameliorate the care of patients.

**Methods:**

We evaluated what MS patients and caregivers perceive as unmet needs and compared patients’ opinions with caregivers’ opinions using a multidimensional questionnaire. The questionnaire was specifically designed for the study, taking into account different aspects of the global care perceived by patients and care givers, such as information about MS, medical treatment and rehabilitation, patients’ relationships with medical staff and their psychological and social life.

**Results:**

We administered the questionnaire to 497 patients and 206 caregivers. Results showed that the majority of participants were satisfied with medical staff but expressed a desire that staff be more forthcoming with information about MS. As for medical treatment concerns, more patients found there to be useful a multidisciplinary approach than caregivers did. Both required psychological support for patients but patients felt a greater need for it at the time of diagnosis, whereas caregivers felt it was required post-diagnosis. Both reported significant strains on patient relationships at work but no effect on other social interactions.

**Conclusions:**

A better understanding of MS patient needs, starting from the point of view of patients and caregivers, could have a great impact on quality of life and on management of the disease.

## Background

MS is a chronic, non-traumatic neurological disease afflicting more than 2.1 million people worldwide [1]; in most cases, MS affects young people of productive age and significantly affects the rest of their lives [2]. MS symptoms may include impaired mobility with limb weakness, poor coordination, sensory problems, vision and hearing loss, cognitive impairment and sphincteric dysfunction. Additional symptoms arise from neuropsychiatric complications [3] with substantial consequences on psychosocial, emotional and working domains [4]. Patients and medical professionals often disagree on issues surrounding health and disease [5]; the concept of “unmet need” is still ambiguous [6]. Several Quality of Life (QoL) studies for MS have shown that patients underscore the emotional problems and burden put on their mental health more than clinicians do [4]. People now acknowledge that psychological, social and psychiatric issues play a major role in health-related QoL [7]. A questionnaire aimed at evaluating caregivers’ QoL has also been proposed recently [8]. Family and caregivers are deeply involved in helping patients cope with the physical and psychosocial effects of the disease. There are some studies that have evaluated the unmet needs of disabled subjects and caregivers, but no tool exists for comparing both patients and caregivers point of view in relation to the same issues, so existing questionnaires were not suitable for the aim of our study. Understanding the needs of patients and what their caregivers perceive to be the unmet needs of patients will increase patient-caregiver satisfaction on a global scale and improve the quality of care [9]. The aim of this study was to evaluate the different perceptions of patients and caregivers about management of MS, particularly about the same items, to gather information to ameliorate the care of patients. Our intention was not to evaluate the impact of the disease on patients’ and caregivers’ QoL by using common instruments, because this could condition our analysis, influencing the answer to the questionnaire subject of the study.

## Methods

### Groups

The study group comprised consecutive participants who attended the MS Centre of the University of Cagliari between October 2010 and July 2011; patients attended the Centre for scheduled visits, to undergo to infusion therapy and to access the rehabilitation service.

Also included in the study are the caregivers. The rationale for including these subjects was to obtain a highly representative sample of MS patients and caregivers regularly attending the clinic. Caregivers were defined as those individuals, connected in various ways to the patients (partners, parents, brothers/sisters, sons, other relatives, professional caregivers and friends), who were dedicated to providing an appropriate and global support of MS patients (physical and psychological).

The exclusion criteria were a refusal to participate, the presence of severe cognitive impairment, [10] vision/hearing disability. The rationale for excluding patients with severe cognitive and/or vision/hearing impairment was that a disability of this nature could influence perceptions of need in a major way. Severe cognitive impairment was evaluated using Rao’s Battery. Only 14 patients were excluded from participation on this basis.

All patients had sufficient vision, hearing and cognitive functioning to complete the survey. The patients with severe motor disability completed the questionnaire with the assistance of a relative (not included in the study). Members of the medical staff approached patients and took informed consent. Patients had needed time to consider to take part to the study. The questionnaire was self-administered, voluntary and filed. We preferred to use an anonymous questionnaire because it included a section regarding satisfaction about medical staff.

All participants completed the questionnaire at the time of inclusion in the study. Patients and caregivers were allowed as much time as needed to complete the questionnaire in a quiet, private and comfortable setting inside the MS clinic.

### Questionnaire design

A multidisciplinary team composed of neurologists, psychiatrists and public health experts developed the questionnaire specifically for the study. In designing the questionnaire, we took into account the opinions of those persons most involved in MS management in our clinic, including therapists, psychologists and nurses. The questionnaire was designed to take into account different aspects of the global care perceived by patients and caregivers.

The questionnaire for patients focused on their level of satisfaction regarding the explanations of current, alternative and future therapies provided by neurologists at the time of diagnosis and afterwards. The second part of the questionnaire focused on patients’ relationships with the neurologists and medical staff, paying particular attention to the requirements of other specialists; this is a very important item because it represents an indirect expression of unmet needs that may be modifiable [11].

The final section asked patients and caregivers to discuss how the disease had affected patients’ personal relationships (with family, friends, partner and colleagues), as well as their psychological state at the time of diagnosis and afterwards.

The items on the questionnaire for patients and caregivers were the same and both focused entirely on the patients’ needs. This was because we wanted to explore the degree of concordance between them.

### All items on the questionnaire were multiple-choice questions

Unmet needs were deduced indirectly, as clearly apparent in the questionnaire. We did not use the expression ‘unmet needs’ in the questionnaire because we suspected that the use of explicit questions could influence the results.

As a preliminary, the two questionnaires were administered to 10 patients and 10 caregivers. Items considered ambiguous, misunderstood or rarely answered were deleted or reworded, while other missing items were added. The preliminary questionnaires were excluded from the analysis.

### Data analysis

Collected data were summarized through descriptive analysis expressing results as percentages and 95% confidence intervals. Differences in perception between patients and caregivers were analyzed with a chi-squared test.

### Ethics

Each participant gave their informed consent for the use of anonymous data for the study. The study was approved by the ethics committee of the University of Cagliari. Data were not nominal at the source and each participant was identified by a code number.

## Results

### Population

The questionnaire was administered at the MS Centre of the University of Cagliari, between October 2010 and July 2011, to 497 MS patients and 206 caregivers. Approximately 200 patients and 100 caregivers did not participate in the study; the principal reason was refusal to participate because of personal organizational problems. Demographic and clinical characteristics were not significantly different between those patients who participated and those who did not. We cannot rule out the possibility that participants attending the clinic may have different needs from those who do not attend, but the aim of the study was to explore different aspects of the global care perceived by patients and caregivers attending the clinic, with the final objective being to support their needs.

The clinical and demographic characteristics of the patient population are summarized in Table 1.

**Table 1:** Patient demographics

**Sex**	**Patients**
Female	319 (64%)
Male	178 (36%)
**Age**	
<20	5 (1%)
21–30	86 (17%)
31–40	151 (30%)
41–50	143 (29%)
>60	112 (23%)
**Onset**	
<5 years ago	143 (29%)
5–10 years ago	177 (36%)
11–20 years ago	120 (24%)
>20 years ago	57 (11%)
**Distance able to walk without rest or assistance**	
Unlimited	322 (65%)
Requires constant assistance to walk 100 meters	
Unilateral assistance	107 (21%)
Bilateral assistance	53 (11%)
Requires wheelchair	15 (3%)

The group of caregivers (206) consists of 35% (72) partners, 28% (58) parents, 17% (35) brothers/sisters, 10% (21) sons, 5% (10) other relatives, 3% (6) professional caregivers and 2% (4) friends; 62% (127) were female and 38% (79) male. There was no difference in caregiver presence on the basis of neurological disability of patients.

### Questionnaire results

#### Diagnosis and conduct of medical staff

Approximately 90% of patients were diagnosed at our Center. We noted that the level of satisfaction regarding diagnosis was high and nearly the same for patients and caregivers. The majority of patients (67%) and caregivers (68%) were completely satisfied. A quarter considered this moment to be lacking in some respects (patients 24%, caregivers 23%) and a minority (patients 9%, caregivers 9%) found it unsatisfactory. As for medical staff conduct, both patients and caregivers considered it kind (patients 64%, caregivers 58%) and attentive (patients 30%, caregivers 34%) (Table 2).

**Table 2:** Satisfaction with diagnosis and medical staff conduct

		**P (tot 497)**	**CI 95% P**	**CG (tot 206)**	**CI 95% CG**	**Chi quadro**	**p value**
	**Completely satisfied**	331 (67%)	62–71	141 (68%)	62–75	0,28	0,871
**Diagnosis**	**Partially satisfied**	120 (24%)	20–28	46 (23%)	17–28		
	**Unsatisfied**	46 (9%)	7–12	19 (9%)	5–13		
	**Kind**	319 (64%)	60–68	120 (58%)	52–65	2,78	0,428
**Opinion of doctor's conduct**	**Attentive**	150 (30%)	26–34	70 (34%)	28–40		
	**Unfriendly**	7 (1%)	0–2	5 (2%)	0–5		
	**Hasty**	21 (5%)	2–6	11 (6%)	2–8		

CI: confidence interval, MS: multiple sclerosis, P: patients, CG: care giver.

#### Source of information

Despite overall satisfaction with how they were diagnosed, the majority of patients (76%) and caregivers (78%) sought more information about the disease, mainly by consulting the Internet (patients 54%, caregivers 53%), media (patients 17%, caregivers 17%) or their personal neurologist (patients 23%, caregivers 25%).

Both patients and caregivers judged the information provided by the doctor about available therapies for MS (patients 79%, caregivers 60%) and therapeutic choices (patients 83%, caregivers 69%) to be exhaustive; a significantly higher degree of patients (*p* = 0.001) found this to be the case. Patients (53%) and caregivers (52%) were equally satisfied with the information provided by the neurologist about future therapy options (Table 3).

**Table 3:** Sources of information and satisfaction with current, future and changes to MS treatment

		**P (497)**	**CI 95% P**	**CG (206)**	**CI 95% CG**	**Chi quadro**	**p value**
	**Needs further information about MS**	376/497 (76%)		160/206 (78%)			
	From neurologist	86 (23%)	19–27	40 (25%)	18–32	4,61	0,595
	From news media	64 (17%)	13–21	27 (17%)	11–23		
	From other MS patients	9 (2%)	1–4	4 (2%)	0–5		
	From other doctor	6 (2%)	0–3	0	0		
	From other neurologist	4 (1%)	0–2	4 (2%)	0–5		
	From friends	4 (1%)	0–2	1 (1%)	0–6		
	From internet	203 (54%)	49–59	84 (53%)	45–60		
**Neurologist explanations for current treatment of MS**	Completely comprehensive	395 (79%)	76–83	124 (60%)	54–67	31,41	0,001
	Partially	84 (17%)	14–20	59 (29%)	22–35		
	Not comprehensive	18 (4%)	2–5	23 (11%)	7–15		
**Neurologist explanations for change of treatment**	Completely satisfied	412 (83%)	80–87	142 (69%)	63–75	16,68	0,001
	Partially satisfied	45 (9%)	6–12	38 (18%)	13–24		
	Unsatisfied	40 (8%)	5–10	26 (13%)	8–17		
**Neurologist explanations for new findings in MS**	Completely satisfied	263 (53%)	49–57	107 (52%)	45–59	0,06	0,971
	Partially satisfied	145 (29%)	25–33	61 (30%)	23–36		
	Unsatisfied	89 (18%)	15–21	38 (18%)	13–24		

CI: confidence interval, MS: multiple sclerosis, P: patients, CG: care giver.

#### Participation in the choice of medical and rehabilitation treatment

More than half of patients and caregivers expressed trust in the therapy (patients 66%, caregivers 55%). It is interesting to note that the majority of caregivers (80%) believed patients were fully involved in therapeutic choices, which was not the case for patients. In fact, about half of the patients (44%) (*p* = 0.001) believed they were not completely invited to participate in choosing a therapy (Table 4).

**Table 4:** Confidence in future therapies and patients participation in choice of treatment

		**P**	**CI 95% P**	**CG**	**CI 95% CG**	**Chi quadro**	**p value**
**Confidence in treatment**	**High**	329 (66%)	62–70	113 (55%)	48–62	8,03	0,005
	**Low**	168 (34%)	30–38	93 (45%)	38–52		
**Participation in choosing a treatment**	**High**	278 (56%)	52–60	164 (80%)	74–85	34,97	0,001
	**Low**	219 (44%)	40–48	42 (20%)	15–26		

CI: confidence interval, MS: multiple sclerosis, P: patients, CG: care giver.

#### Multidisciplinary approach to MS

The second part of the questionnaire focused on consulting other specialists (particularly physiatrists, psychiatrists and urologists). Patients (30%) reported a desire for a multidisciplinary approach (seeing more than one MS specialist), a significantly higher percentage than for caregivers (0) (*p* = 0.001) (Table 5).

**Table 5:** Need to consult other MS specialists l

	**P**	**CI 95% P**	**CG**	**CI 95% CG**	**Chi quadro**	**p value**
**Further needs about medical staff**	307/497 (62%)		87/206 (42%)			
More than one specialist	91 (30%)	25–35	0	0	35,32	0,001
Physiatrist	65 (21%)	17–26	32 (37%)	27–47		
Psychiatrist	51 (17%)	12–21	17 (20%)	11–28		
Urologist	42 (14%)	10–18	17 (20%)	11–28		
Other	58 (18%)	25–35	21 (23%)	15–33		

CI: confidence interval, MS: multiple sclerosis, P: patients, CG: care giver.

#### Social impact of the disease

The last section of the questionnaire, which focused on the disease’s impact (modification over time) on patients’ personal relationships and social interactions, was designed in four parts and based on the different kinds of relationship: with relatives, friends, partners and work colleagues. Patients and caregivers did not significantly differ in their opinions of relationships with friends (patients 74%, caregivers 62%) and family (patients 64%; caregivers 57%), which they generally report to be unchanged. They did have a different perception of the effect on relationships with partners, however; the majority of patients considered it to be unchanged (59%), compared with 42% of caregivers (*p* = 0.001). However, patients (50%) reported a negative effect on their relationships with work colleagues more frequently than caregivers did (43%) (*p* = 0.001) (Table 6).

**Table 6:** MS impact (modification over time) on personal relationships

**Social relationship after diagnosis**	**P**	**CI 95% P**	**CG**	**CI 95% CG**	**Chi quadro**	**p value**
**Relationships with family**						
Did not change	320 (64%)	60–69	118 (57%)	51–64	4,20	0,123
Improved	96 (20%)	16–23	42 (20%)	15–26		
Got worse	81 (16%)	13–20	46 (23%)	17–28		
**Relationships with friends**						
Did not change	367 (74%)	70–78	127 (62%)	55–68	10,41	0,005
Improved	54 (11%)	8–14	34 (16%)	11–22		
Got worse	76 (15%)	12–18	45 (22%)	16–27		
**Relationships with partner (P)**	462		206			
Did not change	272 (59%)	54–63	87 (42%)	35–49	16,34	0,001
Improved	87 (19%)	15–22	59 (29%)	22–35		
Got worse	103 (22%)	18–26	60 (29%)	23–35		
**Relationships with colleagues at working (P)**	365/497		206			
Did not change	168 (46%)	41–51	85 (41%)	35–48	22,77	0,001
Improved	16 (4%)	2–6	33 (16%)	11–21		
Got worse	181 (50%)	44–55	88 (43%)	36–49		

CI: confidence interval, MS: multiple sclerosis, P: patients, CG: care giver.

#### Psychological support

The questionnaire distinguished the need for psychological support at diagnosis and later, over the course of the disease. At diagnosis, both patients (70%) and caregivers (85%) affirmed in equal measure the patients’ need for psychological support, especially from family (patients 21%, caregivers 22%). After diagnosis, however, patients (65%) and caregivers (45%) disagreed about the need for psychological support. Patients (19%) considered familial support less necessary than caregivers (49%) did (*p* = 0.001) (Table 7).

**Table 7:** Need for psychological support at time of diagnosis and post diagnosis

	**P**	**CI 95% P**	**CG**	**CI 95% CG**	**Chi quadro**	**p value**
**Need for psychological support at time of diagnosis**	351/497 (70%)		176/206 (85%)			
Family	72 (21%)	16–25	39 (22%	16–28	8,89	0,114
Psychologist	30 (9%)	6–11	7 (4%)	1–7		
Partner	20 (6%)	3–8	15 (9%)	4–13		
Friends	9 (3%)	1–4	1 (1%)	0–6		
Family doctor	9 (3%)	1–4	2 (1%)	0–3		
Other	203 (58%)	55–65	110 (63%)	57–71		
**Need for psychological support post diagnosis**	325/497 (65%)		92/206 (45%)			
Family	63 (19%)	15–24	45 (49%)	39–59	70,61	0,001
Psychologist	29 (9%)	6–12	13 (14%)	7–21		
Partner	37 (11%)	8–15	21 (23%)	14–31		
Friends	9 (3%)	1–5	4 (4%)	0–9		
Family doctor	5 (2%)	0–3	2 (2%)	0–7		
Other	182 (56%)	51–61	7 (8%)	2–13		

CI: confidence interval, MS: multiple sclerosis, P: patients, CG: care giver.

## Discussion

MS and its treatment significantly affect all aspects of life for individuals at every stage of the disease, regardless of the level of disability, as well as the lives of caregivers. As is widely known, the support of caregivers is very important for MS patients to lead a normal life [12]. Both patients’ and caregivers’ opinions are relevant to understanding which of the patients’ needs are not being met and what can be done to enhance the quality of care. An open dialogue between physician, patients and caregivers can establish a relationship to improve patients’ wellbeing indirectly. Recent findings suggest that some factors, such as acquiring information on MS and communicating with medical staff, can compensate for the worsening of QOL [11]. Acquiring information on MS is useful for psychological adjustment in MS patients since awareness and coping may be associated with this improvement [13].

The increased availability of sources of disease information arise the important issue of quality–not quantity–of information on the disease for MS patients. Nowadays patients may have easily access to unfiltered information on the internet regarding MS, not always being able to interpret them. Therefore the consequences of searching for health information can be both positive and negative [14].

However, a clear discussion with their own neurologists may be more effective in order to obtain a deeper knowledge [15].

The present study is the first step toward a larger project to assess the perspectives of both patients and caregivers. For this purpose, we first ideated a questionnaire that gathers evidence of how patients and caregivers regard the amount of information they receive about MS, the viability of a multidisciplinary approach, their relationships with medical staff, the impact of the disease on patients’ social life and the patients’ need for psychological support. The items on the questionnaires for patients and caregivers were the same and focused entirely on patients’ needs, so that the questionnaires could be useful for us to explore the concordance between them.

Very few research studies about MS patients take into account both patients’ and caregivers’ points of view. Koopman et al. [16] conducted an analogous study with a quantitative questionnaire administered to 353 MS patients and 240 significant others. Despite different styles, questions, items and participating demographics, our questionnaires yielded similar results. This study identified the 10 most important needs for both groups, but there was also a high demand for information regarding MS and psychosocial support (i.e. good relationships with physicians, MS healthcare team, family and friends). Our questionnaire was ideated to reflect the points of view of patients and caregivers about unmet needs related to the disease; no studies are available that evaluate and compare opinions of both. Although it is not a validated tool, it could be useful to obtain patients’ and caregivers’ opinions about the level of satisfaction of our MS management and to explore unmet needs in a clinical practice setting. The questionnaire, which includes a section regarding satisfaction with medical staff, was ideated to be anonymous so as not to affect the answers of participants completing it in the same clinic where the medical staff worked. Results showed a particularly high level of satisfaction on the part of both patients and caregivers regarding neurologists and their way of providing information about diagnoses and treatments. Given that approximately 90% of the patients interviewed were diagnosed at our Center, and because our facility is dedicated exclusively to the diagnosis and care of this disease, it is crucial to understand where we excel (in terms of patient satisfaction) and areas in which we could improve (responding to their expressed dissatisfaction).

In order to achieve a good therapeutic relationship is essential to build a good relationship between the physician and the patient and to listen carefully to patients' descriptions [15]. This explains why we chose to include queries about the staff's aptitude and how they behaved towards patients.

Both patients and caregivers considered our medical staff to be attentive and kind. Both patients and caregivers deemed exhaustive the information provided at the time of diagnosis and afterwards by the neurologists to patients about available therapies for MS and the therapeutic choices. However, it was interesting to note that caregivers believed patients were more involved in choosing their therapy than patients did. In fact, about half of the patients complained about not being completely invited to participate in such decisions. Patients’ desire to play a more active role in their therapy is probably due to their greater emotional involvement.

Despite the fact that patients and caregivers considered the information given by neurologists to be exhaustive, both sought further information, primarily consulting media and Internet sources. This datum, analogous to those found in several other studies, seems to be ‘universal’ [17-20] and points to the gap between MS patients’ perceived need for information and the information they are actually given by key professionals. Moreover, similar findings have been detected in studies on other severe chronic diseases, demonstrating how much patients desire to find understandable information [21]. The unpredictable, variable nature of MS and the possibility of increasing disabilities mean that from the time of diagnosis onward, patients may suffer severe psychological problems.

MS may cause several problems, both visible and invisible. Patients with MS can display multiple objective symptoms related to different systems or less visible problems related to the autonomic nervous system, cognition, mood, pain and fatigue [16]. Symptoms vary between MS patients, and caregivers may only be able to observe them partially. Optimal management of MS symptoms requires a comprehensive multimodal and case-by-case approach [17]. MS patients preferred to see several specialists to treat the disease (in particular physiatrists, psychiatrists and urologists), while caregivers seemed to consider such an approach less important because of their partial understanding of the various aspects of the disease and their focus on contingent problems.

Patients and caregivers agreed upon the need for psychological support, especially from the patients’ families, but patients stressed the importance of such support at the time of diagnosis, whereas caregivers found it was useful at a later stage in the therapy. This finding underscores the sensibility of caregivers regarding the changes in patients’ relationships over the course of the disease. Caregivers’ opinions about the quality of patients’ personal relationships has been found to have an impact on clinical outcome, suggesting that caregivers should be involved in more aspects of patient care [22]. Our study did not take into account the need for psychological support on the part of caregivers, although such a study could be very interesting. Considering treatment for caregivers may be useful to improve overall satisfaction for all parties [23].

## Conclusions

Given the complex physical, psychological and personal impact of MS on patients’ and caregivers’ lives, it is very useful to identify what patients and caregivers perceive to be unmet needs and to compare their opinions. This kind of approach could give us important feedback concerning the quality of care provided and allow us gather information to improve the management of MS.

## Abbreviations

MS: Multiple sclerosis; QoL: Quality of life.

## Competing interests

The authors declare that they have no competing interests.

## Authors’ contributions

LL, MGM and EC participated in the design and coordination of the study and the acquisition and analysis of the data, and drafted the manuscript. GF and GC participated in the acquisition and analysis of the data and drafted the manuscript. CS and GM participated in the analysis of the data and drafted the manuscript. JF, GC and MM drafted the manuscript. All authors read and approved the final manuscript.

## Pre-publication history

The pre-publication history for this paper can be accessed here:

http://www.biomedcentral.com/1471-2377/13/177/prepub
